# Simvastatin Improves Myocardial Ischemia Reperfusion Injury through KLF-Regulated Alleviation of Inflammation

**DOI:** 10.1155/2022/7878602

**Published:** 2022-01-11

**Authors:** Tingju Wei, Jun Li, Guowei Fu, Hui Zhao, Chen Huang, Xiaohua Zhu, Gongcheng Huang, Jing Xu

**Affiliations:** Department of Cardiovascular Surgery, The First Affiliated Hospital of Zhengzhou University, Zhengzhou, China

## Abstract

**Objective:**

To clarify the protective effect of simvastatin on myocardial ischemia reperfusion injury (MIRI) and the underlying mechanism.

**Materials and Methods:**

The MIRI model in rats was firstly constructed. Twenty-four male rats were randomly assigned into the sham group, ischemia-reperfusion (I/R) group, and simvastatin group, with 8 rats in each group. Contents of superoxide dismutase (SOD) and malondialdehyde (MDA), as well as serum levels of CK and inflammatory factors, in rats were determined by the enzyme-linked immunosorbent assay (ELISA). Lactate dehydrogenase (LDH) activity in the three groups was examined. Through flow cytometry and Cell Counting Kit-8 (CCK-8) assay, apoptosis and viability in each group were detected, respectively. Relative levels of HMGB1, Kruppel-like factor 2 (KLF2), eNOS, and thrombomodulin (TM) were finally determined.

**Results:**

Simvastatin treatment markedly enhanced SOD activity and reduced contents of MDA, LDH, and creatine kinase (CK) in MIRI rats. The increased apoptosis and decreased viability following MIRI were partially reversed by simvastatin treatment. Besides, MIRI resulted in the upregulation of inflammatory factors and chemokines. Their elevations were abolished by simvastatin. In MIRI rats, simvastatin upregulated KLF2 and p-eNOS.

**Conclusions:**

Simvastatin protects inflammatory response at post-MIRI through upregulating KLF2, thus improving cardiac function.

## 1. Introduction

Myocardial ischemia reperfusion injury (MIRI) is a cardiovascular disease manifested as acute angina owing to stenosis of the coronary artery and myocardial blood supply disorder [1]. MIRI occurs following reperfusion in thrombolytic therapy, coronary angioplasty, organ transplantation, aortic occlusion, or cardiopulmonary bypass [2]. Currently, it is believed that oxidative stress, intracellular calcium overload and hypercontraction, endothelial cell activation, microvascular dysfunction, and myocardial metabolic changes are major pathogenic factors of MIRI [3]. Eventually, cardiomyocytes are necrotic because of ischemia in myocardial tissues. Researches on alleviating MIRI have been well concerned.

Statins (HMG-CoA reductase inhibitors) are a class of cholesterol-lowering drugs used for preventing and treating cardiovascular diseases [4]. In addition, statins also exert cardioprotective properties through alleviating inflammatory responses [5, 6] and improving endothelial functions [7, 8]. Because of the low cost, statins are extensively applied for the treatment of coronary heart diseases.

Kruppel-like factor 2 (KLF2) is a transcription factor induced by laminar flow in vascular endothelial cells, which is a vital regulator in vascular endothelial functions [9–11]. KLF2 participates in the regulation of inflammation, angiogenesis, barrier integrity, vascular reactivity, and cell phenotypes of the endothelium [12]. Through downregulating eNOS, thrombomodulin (TM), HO-1, and adhesion molecules, KLF2 protects the stability and function of the vascular endothelium from external stimuli [13, 14]. In liver ischemia-reperfusion (I/R) injury, statins inhibit oxidative stress and inflammatory response by upregulating KLF2 [15]. In this paper, we first constructed the MIRI model in rats. The protective role of simvastatin in MIRI was specifically explored.

## 2. Materials and Methods

### 2.1. Experimental Animals

A total of 24 male Wistar rats (250-300 g) were habituated in a standard environment with free access to food and water. Animal procedures were conducted after 12 h food fasting. Rats were sacrificed at 6 and 24 h after reperfusion. This experiment was approved by the Ethic Committee of Zhengzhou University.

### 2.2. MIRI Construction

24 male rats were randomly assigned into the sham group, I/R group, and simvastatin group, with 8 in each. Mechanical ventilation was conducted initially with 5 mL of tidal volume and 50 beats/min of respiratory rate. The rat heart was exposed through left thoracotomy at the third rib space. A 6.0 silk nontraumatic suture was passed through the epicardial layer around the main branch of the left coronary artery, with approximately 2 mm from its starting point. A plastic button with a diameter of about 5 mm was passed through the ligature and in contact with the heart. Both ends of the suture were passed through an exposed small vinyl tube. This method was convenient for generating MIRI in advanced preconditioning (24 hours postoperatively and acute pretreatment). ECG was performed during the whole process of model construction for monitoring cardiac function. Rats in the sham group were subjected to 150 min continuous perfusion. Rats in the I/R and simvastatin groups underwent 30 min ischemia, followed by 120 min reperfusion. Rats in the simvastatin group and I/R group were administrated with 1 mg/kg simvastatin (MedChemExpress, Monmouth Junction, NJ, USA) or 0.1% dimethyl sulfoxide (DMSO) 0.5 h prior to reperfusion, respectively. At 24 h following MIRI, rats were anesthetized for reocclusion of the coronary artery at the initial occluded location, and the heart was collected.

### 2.3. Lactate Dehydrogenase (LDH) Activity Determination

Myocardial tissues were extracted after MIRI, homogenized, and cultured. The culture medium was collected for LDH activity determination using the commercial kit (Jiancheng, Nanjing, China). Values at 450 nm were recorded using a microplate reader.

### 2.4. Enzyme-Linked Immunosorbent Assay (ELISA)

Heart tissues were homogenized in phosphate-buffered saline (PBS) (1 : 10 *w*/*v*) containing 1% Triton X-100 and protease inhibitor [16]. Samples were centrifuged at 4°C and 14000 rpm for 20 min [17]. The supernatant was collected for measuring corresponding indicators using the ELISA kit (Signosis, Santa Clara, CA, USA).

### 2.5. Flow Cytometry

Homogenized tissues were centrifuged at 1000 rpm for 5 min, and the precipitant was harvested and centrifuged again. Cells were resuspended in 100 *μ*L of marker buffer for 15 min. Subsequently, cells were incubated with SA-FLOUS and incubated in dark, at 4°C for 20 min. Absorbances at 488 nm (excitation wavelength), 515 nm (FITC wavelength), and 560 nm (PI wavelength) were recorded for calculating the apoptotic rate.

### 2.6. Cell Counting Kit-8 (CCK-8)

Cells were inoculated in a 96-well plate with 3000 cells per well. At the appointed time points, absorbance value at 450 nm of each sample was recorded using the CCK-8 kit (Dojindo Laboratories, Kumamoto, Japan) for plotting the viability curves.

### 2.7. Quantitative Real-Time Polymerase Chain Reaction (RT-qPCR)

RNAs were extracted from rat myocardial tissues using TRIzol (Invitrogen, Carlsbad, CA, USA) and reversely transcribed into complementary deoxyribose nucleic acids (cDNAs) (Thermo Scientific, Waltham, MA, USA). QRT-PCR was conducted by the SYBR Green method with *β*-actin as the internal reference. Primer sequences were as follows: HMGB1: 5′-TATGGCAAAAGCGGACAAGG-3′ (F) and 5′-CTTCGCAACATCACCAATGGA-3′ (R); KLF2: 5′-GAGCCTATCTTGCCGTCCTT-3′ (F) and 5′-AGCACGCTGTTTAGGTCCTC-3′ (R); eNOS: 5′-CAACTGGAAAAAGGCAGCCC-3′ (F) and 5′-AAGAGCCTCTAGCTCCTGCT-3′ (R); TM: 5′-CCTTTGTCTTTCCGGGCTCT-3′ (F) and 5′-TCAAGTCCTCCCTACCCTCG-3′ (R); and *β*-actin: 5′-TGCTATGTTGCCCTAGACTTCG-3′ (F) and 5′-GTTGGCATAGGTCTTTACGG-3′ (R).

### 2.8. Western Blot

Tissues were homogenized for extracting proteins. After concentration determination, protein samples were loaded on polyvinylidene fluoride (PVDF) membranes (Roche, Basel, Switzerland). Subsequently, nonspecific antigens were blocked with 5% skim milk for 2 h. Membranes were then incubated with primary and secondary antibodies. Band exposure and grey value analysis were finally conducted.

### 2.9. Statistical Analyses

Statistical Product and Service Solutions (SPSS) 22.0 (IBM, Armonk, NY, USA) was used for all statistical analyses. Data were expressed as mean ± SD (standard deviation). Comparison between multiple groups was done using the one-way ANOVA test followed by the post hoc test (least significant difference). *p* < 0.05 indicated the significant difference.

## 3. Results

### 3.1. Simvastatin Alleviated Oxidative Stress at Post-MIRI

**Figure 1:**
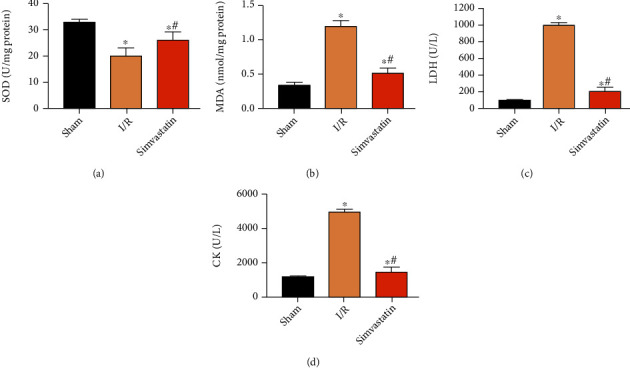
Simvastatin alleviated oxidative stress at post-MIRI. Rats were assigned into the sham group, I/R group, and simvastatin group. Relative levels of SOD (a), MDA (b), LDH (c), and CK (d) in each group. ^∗^*p* < 0.05*vs.* sham group; ^#^*p* < 0.05*vs.* I/R group.

Compared with the sham group, superoxide dismutase (SOD) activity declined and malondialdehyde (MDA) level was elevated in the I/R group. The above changes were markedly reversed by simvastatin treatment (Figures 1(a) and 1(b)). Subsequently, serum levels of LDH and CK were determined in each group. Their elevated levels following MIRI were markedly reduced by simvastatin (Figures 1(c) and 1(d)).

### 3.2. Simvastatin Alleviated Apoptosis at Post-MIRI

**Figure 2:**
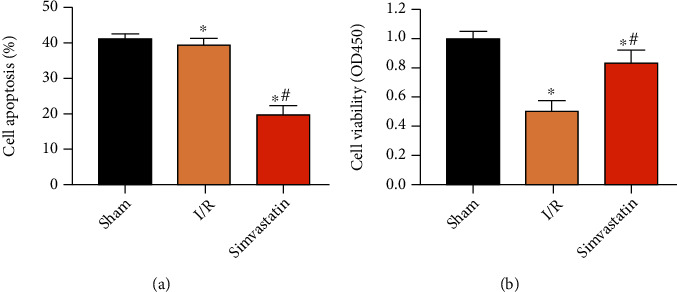
Simvastatin alleviated apoptosis at post-MIRI. Rats were assigned into the sham group, I/R group, and simvastatin group. Cell apoptosis (a) and viability (b) in each group. ^∗^*p* < 0.05*vs.* sham group; ^#^*p* < 0.05*vs.* I/R group.

Cell apoptosis is a key event during cardiomyocyte loss and cardiac insufficiency following MIRI. Our findings uncovered that increased apoptotic rate (Figure 2(a)) and decreased viability (Figure 2(b)) were improved by applying simvastatin.

### 3.3. Simvastatin Alleviated Inflammation at Post-MIRI

**Figure 3:**
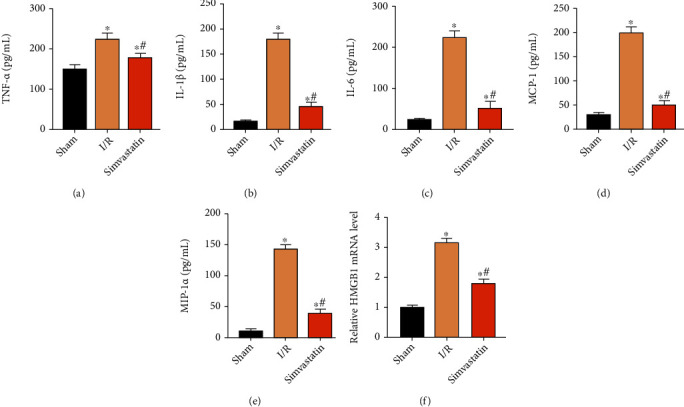
Simvastatin alleviated inflammation at post-MIRI. Rats were assigned into the sham group, I/R group, and simvastatin group. Relative levels of TNF-*α* (a), IL-1*β* (b), IL-6 (c), MCP-1 (d), MIP-1*α* (e), and HMGB1 (f) in each group. ^∗^*p* < 0.05*vs.* sham group; ^#^*p* < 0.05*vs.* I/R group.

Hypoxia triggers the deterioration of inflammatory response [18, 19]. Accumulation of activated inflammatory factors would result in adhesion of neutrophils, further aggravating tissue and organ damage resulted by MIRI [20–22]. Here, increased contents of TNF-*α* (Figure 3(a)), IL-1*β* (Figure 3(b)), and IL-6 (Figure 3(c)) were inhibited by simvastatin. In addition, lower levels of MCP-1 (Figure 3(d)) and MIP-1*α* (Figure 3(e)) were observed in the simvastatin group than in the I/R group. HMGB1 level was also reduced after simvastatin treatment (Figure 3(f)).

### 3.4. Simvastatin Upregulated KLF2 and p-eNOS

**Figure 4:**
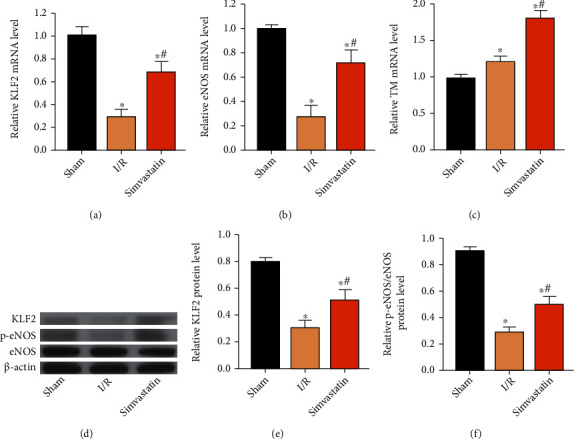
Simvastatin upregulated KLF2 and p-eNOS. Rats were assigned into the sham group, I/R group, and simvastatin group. (a–c) Relative levels of KLF2 (a), eNOS (b), and TM (c) in each group. (d) Western blot analyses on KLF2, p-eNOS, and eNOS in ach group. (e, f) Protein levels of KLF2 (e) and p-eNOS/eNOS (e) in each group. ^∗^*p* < 0.05*vs.* sham group; ^#^*p* < 0.05*vs.* I/R group.

A previous study has shown that simvastatin protects against liver I/R injury through upregulating KLF2 [23]. Here, both mRNA and protein levels of KLF2 were upregulated by simvastatin, which were initially downregulated following MIRI (Figures 4(a), 4(d), and 4(e)). Besides, relative levels of p-eNOS and TM were also upregulated by simvastatin (Figures 4(b), 4(c), and 4(f)).

## 4. Discussion

MIRI is a pathological state with initial limitation on myocardial blood supply and subsequently reperfusion of blood flow [18]. Although reperfusion is the most effective approach for rescuing the ischemic myocardium, it may paradoxically aggravate or cause additional myocardial injury. During the process of MIRI, a series of inflammatory response, oxidative stress, and cytokine activation occur [24]. ROS would be abundantly produced and accumulated in the initial phase of MIRI [25]. Our findings consistently found decreased SOD level and increased MDA level in the I/R group compared to the sham group, suggesting the severe oxidative stress. Notably, simvastatin treatment markedly improved oxidative stress indicators. Changes in hemodynamic parameters support MIRI injury [26]. Herein, relative levels of LDH and CK were determined. Simvastatin markedly reduced their elevated levels following MIRI.

Cell apoptosis is a determinant event for MIRI-induced myocardial infarction [27]. It is reported that inhibition of myocardial apoptosis could reduce 50-70% infarcted size in the myocardium, thus improving cardiac function [28]. In this paper, the apoptotic rate markedly increased, while viability decreased at post-MIRI, and the above trends were abolished by simvastatin.

Inflammatory response following MIRI is of significance in aggravating its secondary injury. MIRI may lead to local aseptic inflammation and production of multiple inflammatory factors [29]. HMGB1 is a vital proinflammatory mediator during the initiation and cascade of inflammation [30]. Simvastatin is demonstrated to prevent inflammation through inactivating inflammatory chemokines [31]. In our analysis, simvastatin markedly downregulated inflammatory factors, chemokines, and proinflammatory mediator in MIRI rats. In addition, KLF2 was downregulated in MIRI rats, and the reduced trend was alleviated by simvastatin treatment. As previously reported, KLF2 is closely linked to the eNOS pathway [23]. In a recent study, it was shown that increased VEGF synthesis in astrocytes is driven by endothelial nitric oxide (NO) generated as a consequence of KLF2- and KLF4-dependent elevation of eNOS in the CCM endothelium [32]. Further, betulinic acid induces eNOS expression via the AMPK-dependent KLF2 signaling pathway [33]. Our results revealed that simvastatin protected the downregulated p-eNOS in MIRI rats. Collectively, we believed that simvastatin protected MIRI through upregulating KLF2. However, the mechanism was very simple; we will further explore the deep mechanism in our next research.

## 5. Conclusions

Simvastatin protects inflammatory response at post-MIRI through upregulating KLF2, thus improving cardiac function.

## Data Availability

The datasets used and analyzed during the current study are available from the corresponding author on reasonable request.
